# canSAR: update to the cancer translational research and drug discovery knowledgebase

**DOI:** 10.1093/nar/gkaa1059

**Published:** 2020-11-21

**Authors:** Costas Mitsopoulos, Patrizio Di Micco, Eloy Villasclaras Fernandez, Daniela Dolciami, Esty Holt, Ioan L Mica, Elizabeth A Coker, Joseph E Tym, James Campbell, Ka Hing Che, Bugra Ozer, Christos Kannas, Albert A Antolin, Paul Workman, Bissan Al-Lazikani

**Affiliations:** Department of Data Science, The Institute of Cancer Research, London SM2 5NG, UK; Cancer Research UK Cancer Therapeutics Unit, The Institute of Cancer Research, London SM2 5NG, UK; Department of Data Science, The Institute of Cancer Research, London SM2 5NG, UK; Department of Data Science, The Institute of Cancer Research, London SM2 5NG, UK; Department of Data Science, The Institute of Cancer Research, London SM2 5NG, UK; Cancer Research UK Cancer Therapeutics Unit, The Institute of Cancer Research, London SM2 5NG, UK; Department of Data Science, The Institute of Cancer Research, London SM2 5NG, UK; Department of Data Science, The Institute of Cancer Research, London SM2 5NG, UK; Department of Data Science, The Institute of Cancer Research, London SM2 5NG, UK; Department of Data Science, The Institute of Cancer Research, London SM2 5NG, UK; Department of Data Science, The Institute of Cancer Research, London SM2 5NG, UK; Cancer Research UK Cancer Therapeutics Unit, The Institute of Cancer Research, London SM2 5NG, UK; Department of Data Science, The Institute of Cancer Research, London SM2 5NG, UK; Department of Data Science, The Institute of Cancer Research, London SM2 5NG, UK; Department of Data Science, The Institute of Cancer Research, London SM2 5NG, UK; Cancer Research UK Cancer Therapeutics Unit, The Institute of Cancer Research, London SM2 5NG, UK; Department of Data Science, The Institute of Cancer Research, London SM2 5NG, UK; Cancer Research UK Cancer Therapeutics Unit, The Institute of Cancer Research, London SM2 5NG, UK

## Abstract

canSAR (http://cansar.icr.ac.uk) is the largest, public, freely available, integrative translational research and drug discovery knowledgebase for oncology. canSAR integrates vast multidisciplinary data from across genomic, protein, pharmacological, drug and chemical data with structural biology, protein networks and more. It also provides unique data, curation and annotation and crucially, AI-informed target assessment for drug discovery. canSAR is widely used internationally by academia and industry. Here we describe significant developments and enhancements to the data, web interface and infrastructure of canSAR in the form of the new implementation of the system: canSAR*black*. We demonstrate new functionality in aiding translation hypothesis generation and experimental design, and show how canSAR can be adapted and utilised outside oncology.

## INTRODUCTION

Since its first release in 2011, canSAR (1–4) continues to be the largest, public, cancer drug discovery resource, used by academia and industry (5) from over 300 countries worldwide. canSAR was originally created to inform target selection for cancer drug discovery. To achieve this goal, we developed canSAR to be a scalable, adaptable and fully integrative knowledgebase. It integrates data from multi-omic profiling of cancer tissue from patients and cancer cell lines, together with data on genetic vulnerabilities and dependencies. These data are fully integrated with vast medicinal chemistry and pharmacology data, annotation of the entire human proteome, protein 3D structure, protein-protein-interactions, drug approvals and clinical trials among other data. The full integration (rather than simple collation) of data means that non-obvious connections can be identified, helping discovery of novel targets and insights for cancer drug discovery and therapy (6–9). We have developed a suite of machine-learning algorithms to learn from these vast integrated data to provide the world's most comprehensive, rapidly updated target druggability/ligandability assessment. These methods assess target feasibility for drug discovery based on 3D structure, known chemistry, behaviour in protein interaction networks, and availability to antibody/biotherapeutics.

Previously, we introduced the first components of the transformation to canSAR (1). Here we describe a complete reimplementation and enhancement of canSAR in a new edition: canSAR*black*. canSAR now contains significant data growth, new web interface and back-end infrastructure, new paradigms for data query and interrogation. Specifically, we are now focusing on developing canSAR to help interpret complex findings and support experimental design.

## DATA IN CANSAR

### Biological data

canSAR contains the entire human proteome (20 375 sequences) from the Uniprot Swiss-Prot (10) database (release 2020_04) as well as >542 000 non-human sequences. canSAR contains a significant increase in all data types, as well as novel data. We have increased the number of molecular profiling studies and now capture multi-omic profiling data on >25 000 cancer patients from large-scale cancer omics initiatives (e.g. TCGA (11), ICGC and Target (12)). Data derive from 94 studies across 26 cancer types, with recent focus being on increasing data on advanced and metastatic, rare and childhood cancers. We perform significant standardisation of the data across studies, curation of the data and annotation with the most appropriate clinical classification systems. For example, although most studies have TNM (TNM Classification of Malignant Tumors) and grade information, we also utilise the Gleason prognostic scores for prostate cancer; FAB, Ann Arbor staging and BINET for blood cancers and FIGO for ovarian cancers etc. The data now include >9 900 000 protein coding mutation data points, >107 million gene-level copy number alterations, >218 million gene expression profiles from tumor samples as well as >194 million normal gene expression profiles (non-cancer donor samples from GTEx (13). In addition to patient profiles, canSAR contains data for ca. 1000 cancer cell lines as well as >25 000 genetic dependency measurements from five different large-scale studies, with >1 186 000 annotated mutations and >22 million gene expression profiles.

### Protein-protein interactions

canSAR contains curated and uniformly assessed protein-protein interaction data that we compile from key interactome databases including the IMEx consortium (14), Phosphosite (15), DePOD (16), HuRI (17), Reactome pathways (18), Tfacts (19), TRRUST (20) and MSigDB (21) as well as key publications (e.g. 22–24). Moreover, we curate protein–protein interactions from the PDB and uniquely identify druggable protein–protein interactions interfaces (see below). In the latest version of canSAR, we have curated >990 000 protein–protein interactions for 18,680 human proteins in Swiss-Prot.

### Drug Combinations

canSAR now includes expert curated and standardised drug combination data for 1456 clinical trials and over 316 000 drug synergies from cancer cell line models from our curated, canSynergize database (drug combination data available for download from cansarblack.icr.ac.uk/resources/cansynergize/cansynergize_latest.zip). This canSAR resource is complementary to other drug combination databases such as DrugComb (25), introducing over 100 novel published cell line-based drug combination studies, as well as exclusive clinical trials-based evidence for drug combinations. It focuses on experimentally observed drug-target associations (through curation of mechanism of action and stringent biochemical assay activity cut offs), and enables simultaneous assessment of drug combination evidence across a large number of targets using the canSAR interactome. canSAR uniquely contains 223 588 drug combination synergy measurements from cellular studies and a further 1456 from clinical trials that are not available in any other public resource.

### 3D structures and druggability assessment

3D protein structure is a key enabler for drug discovery, but also for understanding the likely impact of molecular aberrations on protein function, and generating hypotheses for disease causation. canSAR maintains a weekly update of 3D structural data from PDBe (26). At the time of writing, canSAR contained >518 000 protein chains from >171 000 PDB structures.

Importantly, canSAR also provides a comprehensive and weekly updated structure-based ligandability assessment. canSAR contains ligandability assessment for >4.5 million cavities of which >180 000 are predicted to be ligandable using our predictor. We also analysed >651 000 cavities on >126 000 protein–protein interfaces and identified >81 000 ligandable protein-protein interfaces. New enhancements to canSAR’s evaluation of ligandable sites enable the identification of secondary, regulatory and allosteric ligandable sites. canSAR identified >10 000 non-primary ligandable sites.

Protein 3D structures are not always available for assessment. To overcome this hurdle, canSAR contains several orthogonal assessments for the suitability of targets for therapeutic use and drug discovery. We provide ligand/chemistry-based assessment for 8310 human targets using the chemical and bioactivity information within canSAR. We also calculate the network-based ‘target-likeness’ (27) for 13 467 human targets; as well as the suitability for antibody therapy for all human proteins and identify 3586 targets to be accessible to such biotherapeutics.

### Chemistry and pharmacology

canSAR integrates data from leading medicinal chemistry databases such as ChEMBL (28) and BindingDB (29). The latest version of canSAR contains data for >2 million compounds from these databases. However, there is a large amount of data that would be useful to the wider drug discovery and cancer research communities that are not covered by these outstanding resources. We now capture an increasing number of clinical candidates, research compounds and chemical probes.

To aid the selection of the best chemical probes for experiments, we link extensively with the Chemical Probes Portal (30) (chemicalprobes.org) for expert-evaluated chemical probes; and also to Probe Miner (31) (probeminer.icr.ac.uk) which provides systematic, data-driven large-scale assessment of chemical probes. Collectively, these data enhance the information available to translational researchers in canSAR. With the additional compounds generated through our curation pipelines, canSAR now contains data for >3 million bioactive small molecules.

### Clinical trials

canSAR has a daily updated stream of data from clinicaltrials.gov, and maps these to drug and disease data inside the resource. At the time of writing, canSAR contained data for >351 000 clinical trials.

## ROLE OF TARGETS IN CANCER

**Figure 1.**
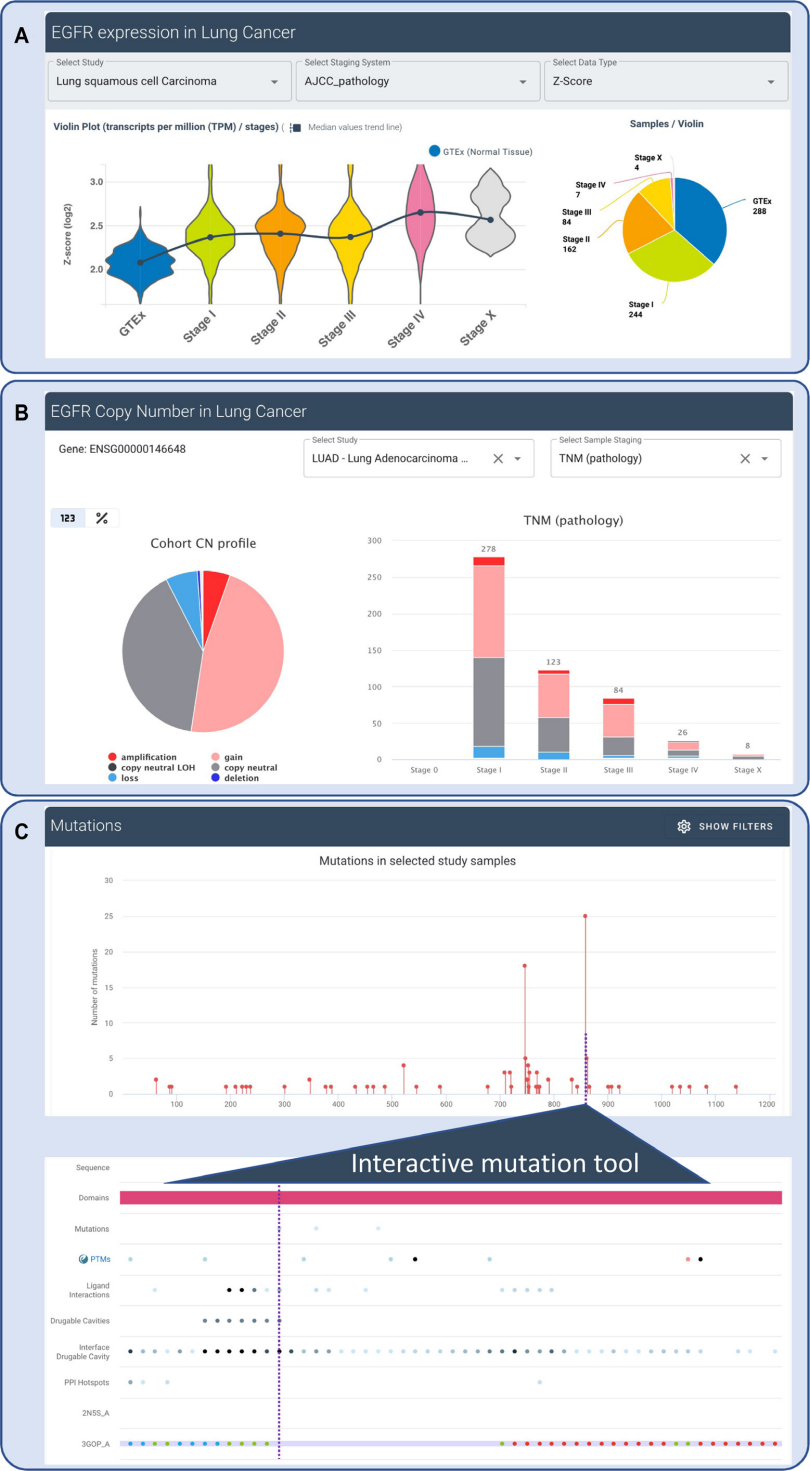
The Cancer Target Association highlights page for EGFR, taken from canSAR. (**A**) Gene expression data comparing EGFR in normal non-cancer samples and the different AJCC pathological stages of lung cancer, showing a gradual increase in EGFR gene expression with progression of the disease; (**B**) Pie chart shows copy number aberrations (CNA) in the TCGA LUAD study, followed by bar charts showing the distribution of different CNAs along pathological stages. The drop-down menu allows users to navigate between studies and cohorts; (**C**) Lollipop plot showing mutation frequency in lung cancer patients along the EGFR amino acid sequence. Users can zoom into a segment of the display to explore this region of the sequence for evidence of functional role. Tracks under the sequence correspond to functional domains, post-translational modifications, ligand/drug binding, protein-interface hotspots, ligandable cavities etc. This interactive tool was developed to enable researchers to generate testable hypotheses for the role of a mutation of interest.

The enhanced target synopsis pages have new and sophisticated functionality to explore potential roles of any human target in cancer. Figure 1 and Supplementary Figure S1 show a subset of the dashboard of information available for EGFR, focusing mainly on novel features and functionality. The user is initially presented with an overview of cancer association assessments for their target of interest, visualised in word cloud form and leveraging both the in-depth pharmacological and molecular disease alteration data in canSAR. The clinical component of the cancer-target association score (CTA), allows the rapid assessment of evidence of approved and investigational drugs targeting the protein of interest, for each cancer type (Supplementary Figure S1A). The molecular alteration components of the CTA score capture the frequency, specificity and effect of alterations (mutations, copy number and expression) for each cancer cohort. A word cloud representation shows cancer types with strongest association with the target can be displayed for individual molecular data types as well as the combination of all alterations.

The user can further explore the cancer-target association evidence by selecting the cancer of their interest from the cancer word cloud. The example shown in Figure 1 and Supplementary Figure S1 is focused on lung cancer. For both approved and investigational drugs targeting EGFR in lung cancer (Supplementary Figure S1B-C), links to the drug chemistry synopsis in canSAR are provided that enable detailed profiling of each compound, together with intervention and clinical trial information. Information on the mutation spectrum, copy number and expression alterations is displayed for patient cohorts pertinent to the selected cancer type, with extensive staging information to allow discovery of potential links to disease progression (Figure 1A, B displaying changes in gene expression and copy number alterations, respectively). Finally, the dependency of the target across a large number of cancer cell lines in systematic genetic screens can be assessed rapidly via a waterfall plot with the cell lines relevant to the selected cancer highlighted in red (Supplementary Figure S1D).

To aid exploration of the likely impact of mutations on protein function, the mutation profile for a target can be interrogated using the interactive mutation tool (Figure 1C). This tool displays the mutation frequency along the protein sequence, aligned with interactive and scalable tracks for evidence of each amino acid in the following areas: position in functional domains; residues known to be post-translationally modified; residues involved in ligand or drug binding; hotspots in protein-protein interfaces; residues participating in ligandable cavities (as assessed by the canSAR 3D ligandability pipeline) belonging to the protein of interest or formed as a result of a direct interaction with a protein partner. The interactive tool additionally selects representative 3D structures to allow the user to display the position of the mutation on the appropriate 3D structure of the target of interest. This tool can help generate hypotheses about the role of a particular mutation in ligand-binding, interference with drug binding, or regulatory activity. Users can navigate between different cohorts and studies, fine tuning their hypothesis generation.

## TECHNICAL FEASIBILITY FOR TARGET VALIDATION AND TRANSLATIONAL RESEARCH

Once a target has been prioritised from a biological evidence perspective, researchers may wish to perform a series of experiments to validate the biological role in the cancer of interest, and potentially explore whether the target could be progressed in drug discovery. Now, through the enhanced functionality and integration of data in canSAR, we are able to identify and provide key information to inform experimental design for mechanistic evaluation and target validation.

**Figure 2.**
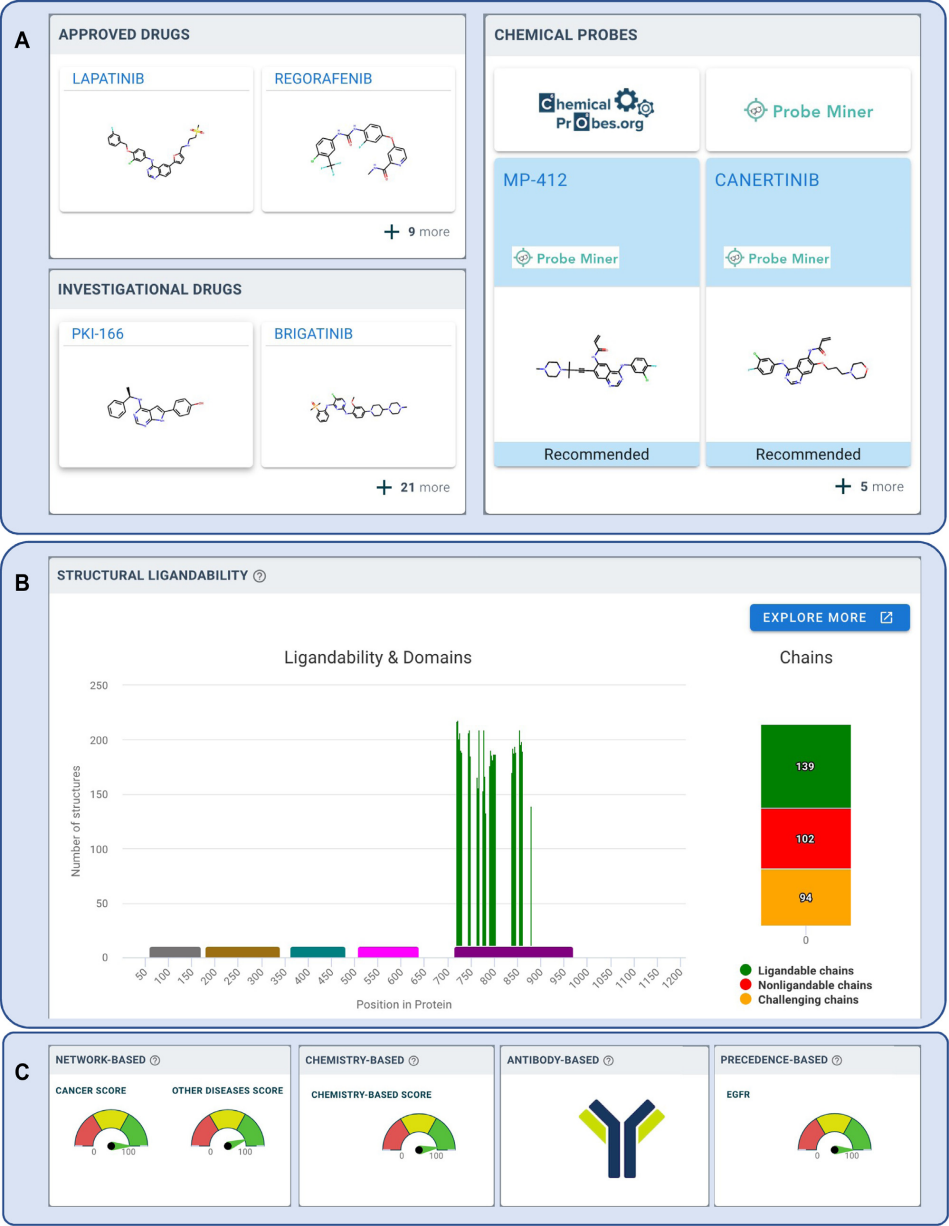
Data supporting technical feasibility assessment for EGFR, taken from canSAR. Analysis of technical feasibility can be explored in terms of target tractability and experimental practicability (Supplementary Figure S2). (**A**) Available drugs – as this is a clinically-precedented drug target, approved drugs and investigational drugs are shown at the top. Where canSAR is able to recommended chemical probes, these are also shown with links to the primary chemical probe resources (Chemical Probes Portal and Probe Miner); (**B**) A summary of the structure-based ligandability. The protein sequence is represented along the x-axis and known functional domains shown on top. A green histogram shows where predicted ligandable cavity positions lie; in this case, illustrating their clustering on the catalytic kinase domain; further details and interactive exploration of protein 3D structures and ligandable cavities can be reached through the ‘explore more’ button; (**C**) Summary of the orthogonal ligandability/target likeness assessments calculated in canSAR. Dials represent the percentile ranking of EGFR in each assessment: network-based assessment determines how closely the target resembles drug targets from oncology or other disease areas; ligand-based assessment assesses a target based on the properties, number and bioactivity of its ligands and bioactive molecules; precedence-based assessment explores family similarity to known drug targets; and finally an indication of whether the target is available to extracellular antibody or other biotherapeutics. These methodologies utilise predictive machine learning algorithms and are calculated for most proteins in the human proteome, thus enabling the assessment of novel, potentially druggable targets.

We have previously described canSAR’s unique suite of machine learning algorithms for the prediction of ligandability of proteins (1). canSAR provides a visual summary of ligandability that is weekly updated. It presents approved drugs, clinical candidates and chemical probes that are curated in canSAR (Figure 2A). A domain representation of the protein sequence is displayed (Figure 2B) with a histogram (in green) showing the concentration of ligandable cavities along the sequence. The example of EGFR in Figure 2B shows that ligandability is clustered around the protein kinase catalytic domain. Figure 2C shows the assessment using canSAR’s orthogonal target-likeness assessments using precedence, protein network behaviour, and accessibility to antibodies.

To support experimental design for target validation or mechanistic studies, canSAR’s 'Experimental Tools' section provides useful information, as detailed below (Supplementary Figure S2).

Chemical probes are powerful tools to explore molecular mechanisms, that are complimentary to genetic manipulation. However, the scientific literature suffers from ubiquitous use of poor quality compounds claimed as chemical probes, see references (30–33). To help inform scientists on appropriate probes we list recommended or acceptable probes for use and link through to further information in the Chemical Probes Portal and Probe Miner (Supplementary Figure S2A).

A key component of mechanistic evaluation and target validation is defining target engagement/modulation biomarkers. As a first step towards biomarker identification, we utilise our linked kinase–substrate residue phosphorylation information (in collaboration with Phosphosite (15); Supplementary Figure S2B). The user can select specific phosphorylation sites on the target itself or on downstream substrates that, to the best of our knowledge, are unique to the target's enzymatic action. Though the biomarker decision is dependent on the specific experimental question being addressed, the user is alerted when additional targets are known to modify the same site and can factor in this information to their experimental design.

To help identify suitable cancer cell lines for experiments, canSAR provides a ranked list of these for consideration by the biologist. The ‘Cell Line Annotations’ module ranks 1300 cell lines based currently on target expression levels in the cell line and evidence of genetic dependency. Genetic dependency utilises the Sanger project SCORE (34) and DepMap CRISPR and RNAi analyses (35). Cancer cell lines are ranked by the degree of their dependency on the specific target. For expression ranking, cell lines are ranked according to the normalised level of expression of the specific target. In addition, cell lines are assessed for sensitivity in functional assays performed with good quality chemical probes and approved / investigational drugs. Significant evidence for each of the three categories assessed is portrayed visually (Supplementary Figure S2C), and combined to generate the overall cell line ranking. The user can explore the complete cancer cell line spectrum and prioritise lines by desired experimental approach (e.g. CRISPR versus chemical target inhibition), be alerted on low or unknown target expression cell lines and informed on cell lines for a specific cancer type.

Finally, we are compiling evidence for the most commonly used expression systems for the production of each human protein target, e.g. for use in biochemical assays and structure determination. We present these as word clouds denoting the most commonly used expression systems (Supplementary Figure S2D).

## UNIQUE CHEMICAL ANNOTATION

A key challenge in the integration of chemical and pharmacological data is that the same compounds may be represented differently in different databases. A key source of this discrepancy is whether stereochemistry is explicitly represented or what tautomer is used to represent the compound. The result of this inconsistency is that often, very important bioactivity data relating to the same particular compound will not be identified because it is tagged against different chemical structure representations and thus different IUPAC International Chemical Identifiers (InChIs). To overcome this challenge, we have implemented a new chemical registration pipeline and chemical grouping system. This enables users to rapidly identify equivalent or highly related chemical structures and explore their bioactivities where appropriate.

To develop our pipeline, we developed a *Knime* workflow using *Knime* version 4.1.3. with *RDKit KNIME integration* version 4.0.1.v202002121352 and *KNIME Python Integration* version 4.1.3.v202005112253 running on a *Python* 3 environment; *MolVS* version 0.1.1, ChemAxon version 20.18.0. Class Tautomerization Plugin was used for canonicalization of molecules, Marvin 20.18, 2020, ChemAxon. Standardizer was used for structure transformation, JChem 20.18.0, 2020, ChemAxon (http://www.chemaxon.com). In summary, compounds from multiple data sources (e.g. ChEMBL (28), BindingDB (29), and canSAR own compounds) are imported and any violations to the SDF format are corrected. Compounds are then Kekulised using *RDKit Kekulizer* and standardised using *MolVS Standardizer* tool. A key change is the subsequent use of *ChemAxon Class Tautomerization Plugin* to generate canonical tautomers of all compounds. Subsequent salt-stripping and tautomer generation of the free base/acid is carried out.

**Figure 3.**
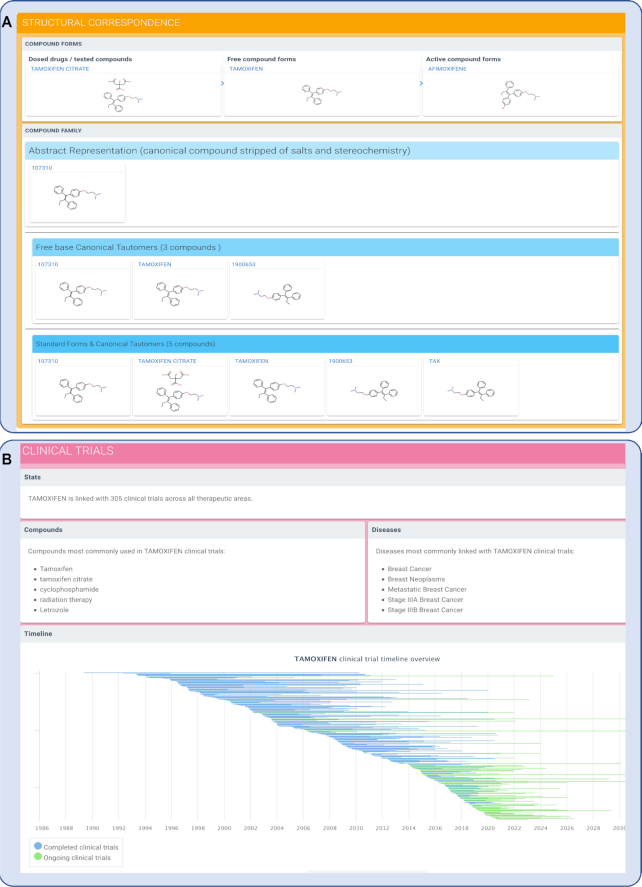
Segments of the new compound synopsis pages, illustrated here for the anti-hormonal breast cancer drug tamoxifen. (**A**) Chemical compound data showing structural correspondence. First, the relationship between the dosed ingredient which is a pro-drug, and the active form of the compound, the main, 4-hydroxy metabolite afimoxifene. Then, the parent compound, stripped of stereochemistry, allows grouping of different enantiomers as well as different salt forms of the compound. The freebase canonical tautomer layer shows three distinct canonical tautomers for all forms of tamoxifen in the group compounds that are chemically equivalent but represented differently in source databases. Moreover, the structure allows the identification of related enantiomers and molecules with undefined stereochemistry. In this case, our pipeline not only identifies R/S stereochemistry but also E/Z stereochemistry, with the three E/Z/undefined alkene stereoisomers shown. While the bioactivity data for these should not be mixed, the display alerts the user to related compounds that may hold useful information; (**B**) Clinical trials information. Users can navigate increasing details of compound information. For example, users can instantly link to clinical trials for the compounds where they exist. An interactive clinical trials view allows users to navigate and filter clinical trials based on disease, phase and other trial characteristics.

This pipeline results in the ability to group compounds with the same canonical tautomer, hence enabling users to identify bioactivity data relating to their particular chemical of interest, regardless of its original representation. Figure 3A shows the structural family for the anti-hormonal drug tamoxifen. First, the relationship between the dosed ingredient which is a pro-drug, and the active form of the compound which is the major, 4-hydroxy metabolite afimoxifene, is displayed. Then, the parent compound, stripped of stereochemistry, allows grouping of different enantiomers. Importantly, the canonical tautomers enable the user to group together compounds that are chemically equivalent but are represented differently in source databases. Moreover, the grouping system facilitates the identification of related enantiomers and molecules with undefined stereochemistry. While the bioactivity data for each of the chemical structures is not be mixed, the system alerts the user to related compounds that may hold useful information. As a result, we now have >3 million bioactive small molecules in canSAR linked through these hierarchies and through to all related bioactivity data, 3D structures and also clinical trials where available.

From this page, users can navigate through increasing details of compound bioactivities and profiles. For example, users can instantly link to clinical trials for compounds, where these exist (Figure 3B). An interactive clinical trials view allows users to navigate and filter clinical trials based on disease, phase and other trial characteristics.

## A NEW UNDERLYING INFRASTRUCTURE

To maintain the power of canSAR and its future compatibility, we re-implemented the underlying infrastructure to use open-source technical components where possible (e.g. PostgresDB, Nuxt.js, Vuetify) that provide a faster, lean product. We leverage Docker to provide a dynamic way to extend our resources only when needed, thus maintaining control of all components. canSAR contains >10 billion integrated experimental and clinical measurements, the power of which is the context and integration. Several searches and data tables can be downloaded for particular user queries such as the interactome pages. Additionally, large batch annotation tools such as the Cancer Protein Annotation Tool allows users to customise large datasets to download. canSAR uses web-development industry consensus best practices to allow for better accessibility for users with impairments, enhanced performance and also provide search engine optimisation (SEO). We follow industry standards regarding colour contrasts, responsiveness of website on various screen sizes and devices; as well as visual separation of components. canSAR provides extensive browser compatibility for all standards-compliant browsers (https://canSAR.icr.ac.uk/#splash-about). It is also implemented for cross-device comparability and adapts for use on mobile devices and low-resolution screens.

The canSAR interface resembles a deep encyclopaedia of information and tools. To help users understand the specific information and visualisations, we have implemented an extensive set of ‘in-line’ tool tips that contain glossaries and explanations.

## BEYOND ONCOLOGY

While we developed canSAR to support cancer research and drug discovery, a key philosophy from the outset was to remain comprehensive in much of the data that we capture, going well beyond oncology. As such, canSAR contains the entire human proteome and all protein sequences in Uniprot (10); 3 million bioactive chemical structures and >8 million bioactivities, the entirety of PDB (26) etc. The reason for this strategy was 2-fold: (i) to ensure that cancer drug discovery benefits maximally from efforts in other therapeutic areas and (2) to support the discovery of novel cancer biology beyond what is already known. As a result, canSAR is already being used by drug discovery communities beyond cancer, especially to make use of the unique druggability and target assessments.

The global COVID-19 pandemic has had a significant impact on cancer research and cancer care. While the international community scrambled to identify potential therapies and develop vaccines, a growing and chaotic cacophony of research data was being produced and published, some with very little scientific validation. This meant that misinformation became rife, and some clinical trials were started without clear mechanistic rationale; meanwhile, valuable research and insights were being missed as they were obscured in the chaos. There was a clear need for an objective, data-driven resource to inform and support the coronavirus drug discovery effort. Due to its comprehensive nature, we were able to rapidly develop a coronavirus edition of canSAR (coronacansar.ac.ac.uk) to help support and inform drug discovery and experimental validation for all coronaviruses. A separate publication preprint describing corona canSAR is available (36).

## CONCLUSION

canSAR has grown significantly as the leading public oncology drug discovery resource. It now contains >10 billion experimental measurements across multiple disciplines, incorporating patient molecular profiling, medicinal chemistry, structural and systems biology and clinical studies. These data benefit from extensive curation, standardisation and insights. The current canSAR release is the most comprehensive, up-to-date and unique provider of target ‘druggability’ and ligandability assessment.

Future developments of canSAR will focus on unique data and interpretive value. We will continue to enhance the data and curation, and to add new data that are not available to cancer researchers through other resources. Crucially, future developments will focus on decision support and experimental guidance for translational research in oncology, while maintaining its disease independent capabilities in target druggability assessment and prioritisation.

## Supplementary Material

gkaa1059_Supplemental_Files

## Notes

Present address: Elizabeth A. Coker, Wellcome Sanger Institute, Hinxton, UK.

Present address: Bugra Ozer, Healx Ltd., Cambridge, UK.

Present address: Christos Kannas, Hit Discovery, Discovery Sciences, R&D AstraZeneca, Gothenburg, Sweden.
