# Supplementary material for: canSAR: update to the cancer translational research and drug discovery knowledgebase
Source: Nucleic Acids Res. 2020 Nov 21;49(D1):D1074–82. doi: 10.1093/nar/gkaa1059 (PMC7778970; doi:10.1093/nar/gkaa1059)
Supplement: gkaa1059_Supplemental_Files [file gkaa1059_supplemental_files.zip › canSAR_NAR_2020_Supplementary_Figures.pptx]

## Slide 1
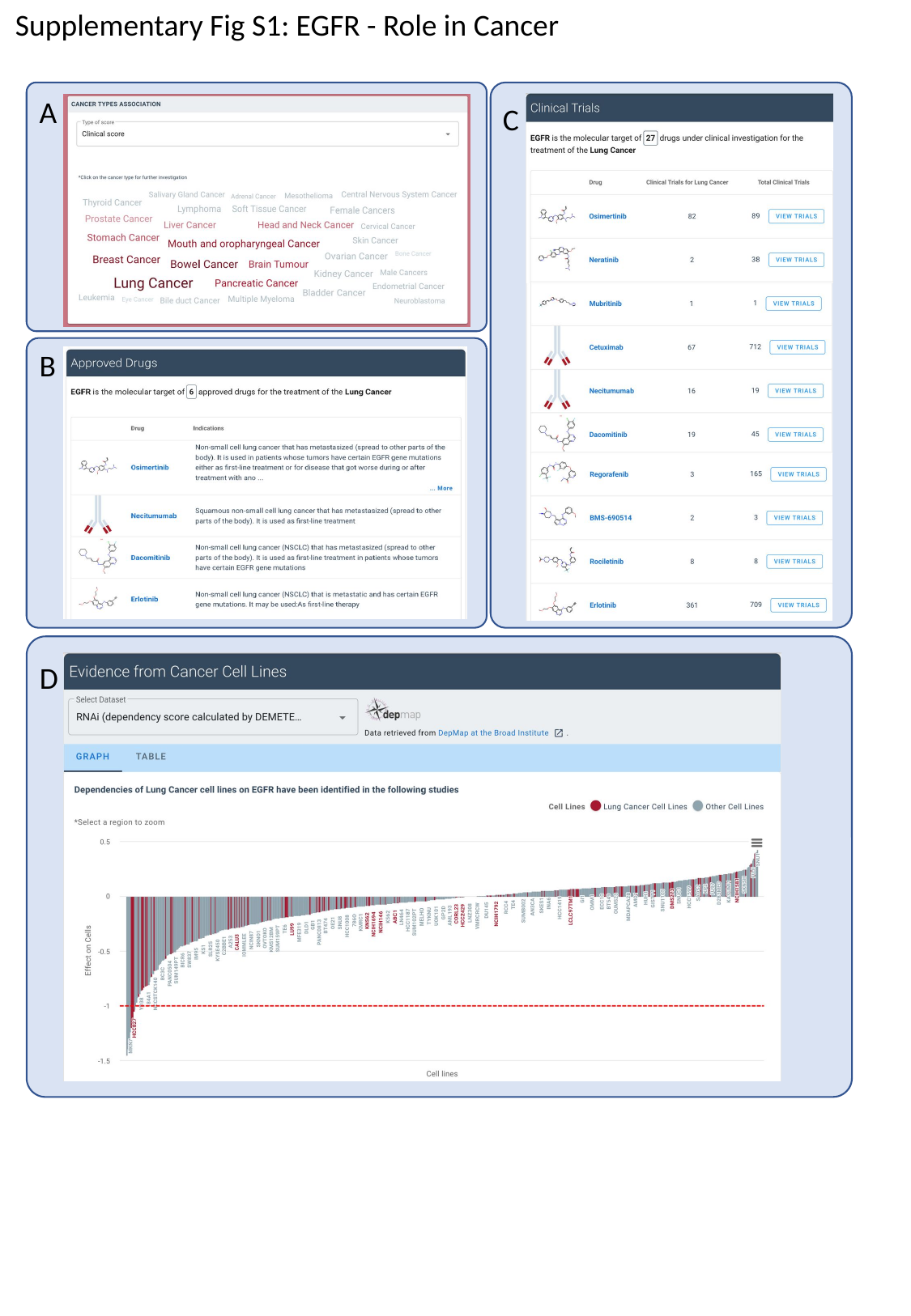

Supplementary Fig S1: EGFR - Role in Cancer
A
C
B
D

## Slide 2
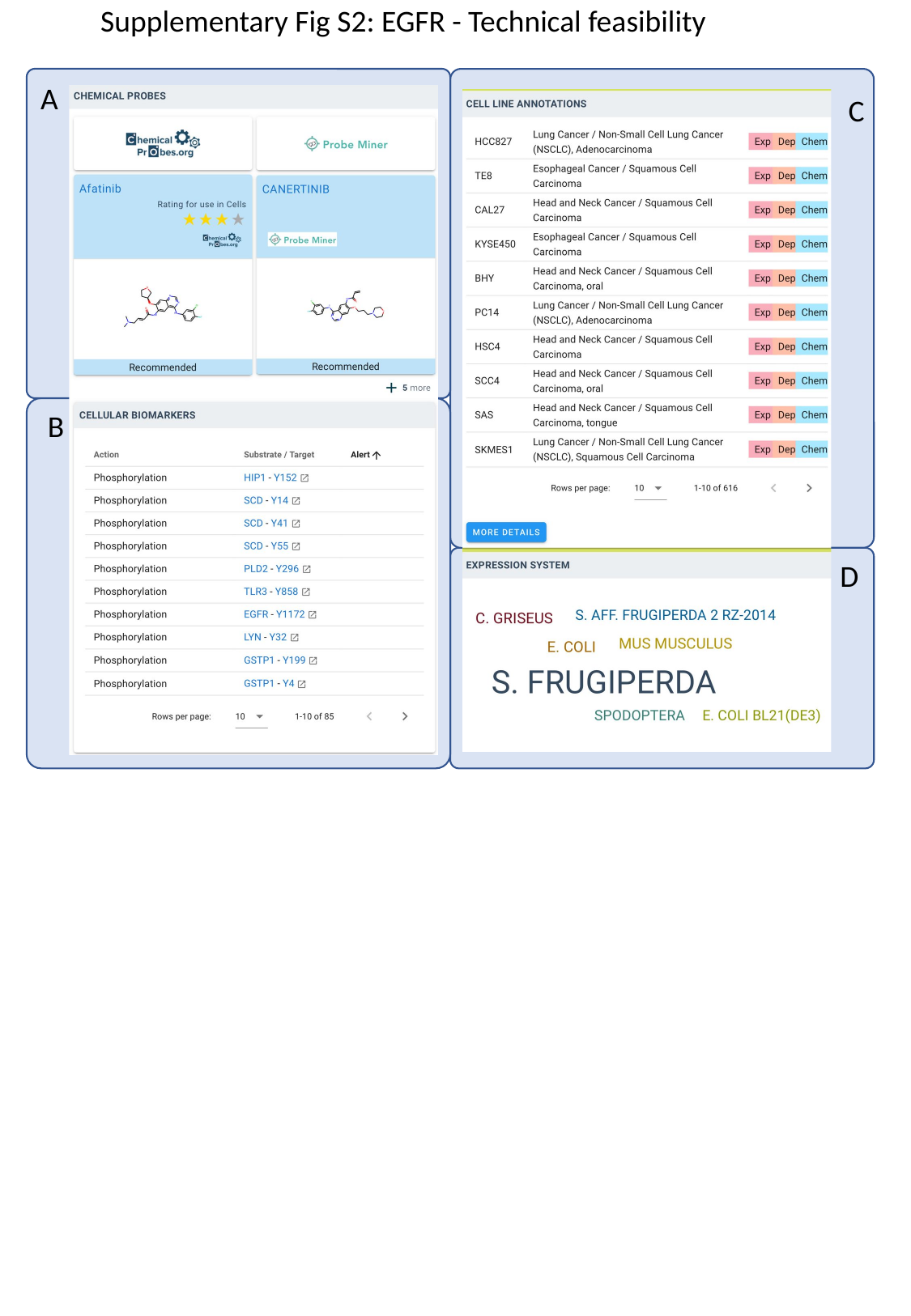

Supplementary Fig S2: EGFR - Technical feasibility
A
C
B
D
