# Supplementary material for: canSAR: update to the cancer translational research and drug discovery knowledgebase
Source: Nucleic Acids Res. 2020 Nov 21;49(D1):D1074–82. doi: 10.1093/nar/gkaa1059 (PMC7778970; doi:10.1093/nar/gkaa1059)
Supplement: gkaa1059_Supplemental_Files [file gkaa1059_supplemental_files.zip › Supplementary Figures.docx]

## Supplementary Figure S1:

The Cancer Target Association highlights page for EGFR, taken from canSAR. A) Word cloud illustrating the association of EGFR with different cancer types, showing that lung cancer is the cancer most closely linked with clinical utility of EGFR; B) List of the approved drugs that are curated in canSAR as both targeting EGFR and approved for lung cancer indications; C) List of the investigational drugs that are curated in canSAR as both targeting EGFR and in clinical trials for lung cancer indications. Note that drug curation is an ongoing activity in canSAR and we aim to complete comprehensive curation of all cancer investigational and key discovery stage drugs in 2020; D) Interactive waterfall plot to explore the effect of genetic manipulation of EGFR in cancer cell lines. The red bars corresponding to lung cancer cell lines. Users can explore this information for every target, in all individual cancer types as well as pan-cancer.

## Supplementary Figure S2

Data supporting technical feasibility assessment for EGFR, taken from canSAR. Analysis of technical feasibility can be explored in terms of target tractability and experimental practicability. Experimental tools, aims to shed a light on the technical feasibility and help inform experimental design; A) Lists of recommended or acceptable chemical probes where available; B) Lists of known phosphosites that could be explored as target engagement biomarkers. If other kinases are known to also phosphorylate these sites, warnings are issued to alert the user to this redundancy. We are now expanding this data set beyond kinase phosphorylation sites; C) Ranked list of cell lines that can be used for investigation of this target. Currently they are ranked based on evidence of target gene expression, genetic dependency and bioactivity of known drugs or chemical probes. Which of these categories were identified as significant in a particular cell line is represented through the appropriate icon. Full details and all cell lines can be explored via the ‘More details’ button; D) Protein expression systems most commonly identified for the target represented as a word cloud with the most common systems showing as largest. All of the above segments are currently under active enhancement and curation.
